# *Riemerella anatipestifer* Type IX Secretion System Is Required for Virulence and Gelatinase Secretion

**DOI:** 10.3389/fmicb.2017.02553

**Published:** 2017-12-19

**Authors:** Yunqing Guo, Di Hu, Jie Guo, Tao Wang, Yuncai Xiao, Xiliang Wang, Shaowen Li, Mei Liu, Zili Li, Dingren Bi, Zutao Zhou

**Affiliations:** ^1^College of Veterinary Medicine, Huazhong Agricultural University, Wuhan, China; ^2^State Key Laboratory of Agricultural Microbiology, Huazhong Agricultural University, Wuhan, China; ^3^Key Laboratory of Preventive Veterinary Medicine in Hubei Province, Huazhong Agricultural University, Wuhan, China

**Keywords:** *Riemerella anatipestifer*, T9SS, *sprT*, virulence, gelatinase secretion

## Abstract

*Riemerella anatipestifer* (RA), a major causative agent of septicemia anserum exsudativa in domesticated ducklings, has a protein secretion system known as the type IX secretion system (T9SS). It is unknown whether the T9SS contributes to the virulence of RA through secretion of factors associated with pathogenesis. To answer this question, we constructed an RA mutant deficient in *sprT*, which encodes a core protein of the T9SS. Deletion of *sprT* yielded cells that failed to digest gelatin, an effect that was rescued via complementation by a plasmid encoding wild-type *sprT*. Complement-mediated killing was significantly increased in the deletion mutant, suggesting that proteins secreted by the T9SS are necessary for complement evasion in RA. Liquid chromatography-tandem mass spectrometry analysis revealed that RAYM_01812 and RAYM_04099 proteins containing a subtilisin-like serine protease domain and exhibiting extracellular gelatinase activity were secreted by the T9SS. Animal experiments demonstrated that the virulence of mutant strain Δ*sprT* strain was attenuated by 42,000-fold relative to wild-type RA-YM. Immunization with the Δ*sprT* protected ducks from challenge with RA-YM, suggesting that the former can be used as a live attenuated vaccine. These results indicate that the T9SS is functional in RA and contributes to its virulence by exporting key proteins. In addition, subtilisin-like serine proteases which are important virulence factors that interact with complement proteins may enable RA to evade immune surveillance in the avian innate immune system.

## Introduction

*Riemerella anatipestifer* (RA) is a Gram-negative, non-spore-forming, rod-shaped bacterium belonging to the genus *Riemerella* and family *Flavobacteriaceae* of the phylum *Bacteroidetes* (Segers et al., 1993). It usually causes septicemia and serum exudate in ducks, geese, turkeys, and various other domestic and wild birds, resulting in serious economic losses worldwide (Sandhu, 2008). To date, 21 serotypes of RA have been identified and no significant cross-protection has been reported (Sandhu and Leister, 1991; Pathanasophon et al., 2002; Sandhu, 2008). Inactivated bacterins have been used in ducks to prevent RA infection. Live avirulent strains induce longer-lasting protection and are more convenient; however, there are few reports on attenuated live vaccines (Sandhu, 1991; Higgins et al., 2000; Liu et al., 2013). Chemotherapy is the most widely used approach for treating RA infection, but the increasing incidence of drug resistance (Yang et al., 2012; Li et al., 2016) compels the search for new strategies for controlling this disease.

Recently, a novel protein secretion system known as the T9SS or Por secretion system associated with gliding motility and secretion of virulence factors was discovered in many species of *Bacteroidetes* (Sato et al., 2010; Mcbride and Zhu, 2013; Ksiazek et al., 2015b; Abby et al., 2016; Lasica et al., 2017). The T9SS has been studied in the motile *Flavobacterium johnsoniae* as well as in the non-motile *Porphyromonas gingivalis* and *Tannerella forsythia*, the latter being the major causative agents of periodontitis. The core set of T9SS genes includes *gldK, gldL, gldM, gldN, sprA, sprE*, and *sprT* in *F. johnsoniae* and their orthologs *porK, porL, porM, porN, sov, porW*, and *porT* in *P. gingivalis*. Deletion of some components of the T9SS resulted in protein secretion defects (Rhodes et al., 2010, 2011; Sato et al., 2010; Shrivastava et al., 2013; Veith et al., 2013). Proteins secreted by the T9SS have an N-terminal signal peptide that enables transit across the cytoplasmic membrane via the Sec system, as well as a conserved CTD that is thought to target the proteins to the T9SS (Sato et al., 2010; Slakeski et al., 2011; Glew et al., 2012; Mcbride and Zhu, 2013). Genes encoding T9SS protein orthologs to those in *F. johnsoniae* and in *P. gingivalis* have been identified in the genome of RA strain RA-YM (*RAYM_04711, RAYM_04706, RAYM_04701, RAYM_04696, RAYM_09602, RAYM_03704*, and *RAYM_03924*) (Zhou et al., 2011). Although the pathogenicity of bacteria is closely related to protein secretion systems, it is unknown whether the T9SS contributes to RA virulence through the secretion of factors associated with pathogenesis or the stress response.

The T9SS component SprT is predicted to be a membrane-associated protein (Sato et al., 2005; Nguyen et al., 2009; Shrivastava et al., 2013). SprT is involved in gliding motility, extracellular chitinase activity, and localization of SprB adhesin in *F. johnsoniae* (Rhodes et al., 2010; Sato et al., 2010). PorT, orthologs to SprT, is essential for the secretion of gingipains in *P. gingivalis* (Sato et al., 2005, 2010; Nguyen et al., 2009). A previous study reported that *sprT* expression was regulated by the iron and ferric uptake regulator Fur, suggesting that the role of SprT protein is to ensure cell survival and fitness by providing transportation to proteins under iron-restricted conditions (Guo et al., 2017). To date, there have been no reports on the T9SS of RA. In this study, we constructed the *sprT* (*RAYM_03924*) gene deletion mutant Δ*sprT* and the complemented strain CΔ*sprT* to investigate the relationship between the T9SS and the biological characteristics and virulence of RA.

## Materials and Methods

### Bacterial Strains, Plasmids, and Culture Conditions

Bacterial strains and plasmids used in this study are listed in **Table 1**. RA-YM was isolated and deposited at the Department of Veterinary Microbiology and Immunology of Huazhong Agricultural University. RA strains were grown on TSA (Difco, Detroit, MI, United States) or TSB (Difco) at 37°C in 5% CO_2_. *Escherichia coli* was grown at 37°C on a Luria-Bertani plate or in Luria-Bertani broth. Antibiotic concentrations were as follows: ampicillin, 100 μg/mL; chloramphenicol, 50 μg/mL; spectinomycin (Spc), 100 μg/mL; and diaminopimelic acid, 50 μg/mL.

**Table 1:** Strains, plasmids, and primers used in this study.

Strains, plasmids, and primers	Descriptions	Source
**Strains**		
RA-YM	*Riemerella anatipestifer* wild-type strain, serotype 1	Preserved in the laboratory
Δ*sprT*	*sprT* gene deletion mutant strain, Spc^R^	This study
CΔ*sprT*	Complemented RA-YM aaa*sprT* strain, Spc^R^, Amp^R^	This study
*E. coli* DH5α	Competent cell	Transgen
*E. coli* X7213	Competent cell	This study
**Plasmids**		
pMD18-T	TA cloning vector	Takara
pRE112	Suicide vector	Preserved in the laboratory
pRE112-LSR	Suicide vector	This study
pRES-JX-bla	Shuttle vector	Preserved in the laboratory
RA-JX	Wild type plasmid of RA	Preserved in the laboratory
pRES-JX-bla-sprT	Shuttle vector	This study
pGEX-6P-1	Expression vector	Preserved in the laboratory
pGEX-6P-1_01812	Expression vector	This study
**Primers (5′–3′)**		
Spc-F1	CAGTGGAACGAAAACTCACGTT	This study
Spc-R1	CAGTAGTTTTAAAAGTAAGCACCTG	This study
SprT-F1	ACGGGTACCAGGTACTAAAGCCGAAAT	This study
SprT-R1	ACGTGAGTTTTCGTTCCACTGCATGG CAGAAATACTAATG	This study
SprT-F2	TGCTTACTTTTAAAACTACTATACAC GCTGATAGATGG	This study
SprT-R2	CGAGCTCCAAACCTCGGTAAACAAA	This study
SprT-IS	ATGGACAGAATGGAAGGC	This study
SprT-IR	TTTTGGAGTGAATGGAGC	This study
Promoter-sprT1	GGGTACCAAGTATTTTGGATAAATTAGACT	This study
Promoter-sprT2	TATCTTTTTCATTAATCTTAAAAATTTACAGCCAC	This study
SprT-CF	TTTTTAAGATTAATGAAAAAGATATTTTTTTTAAC	This study
SprT-CR	GGCATGCTTATTCAAATTTTAGCACAAACA	This study
P01812-1	CGGGATCCATGGCTCAAAACCAAAACACATC	This study
P01812-2	CCCTCGAGCTTCTTGATAAATTTCTTAGTAA	This study

*^R^Resistance.*

### Construction of Mutant Strain Δ*sprT* and Complemented Strain CΔ*sprT*

The mutant strain Δ*sprT* was constructed by allelic exchange using the recombinant suicide plasmid pRE112. Briefly, a 585-bp left flanking region of the *sprT* gene (Leftarm) was amplified from RA-YM genomic DNA using primers SprT-F1 (introducing a *Kpn*I site) and SprT-R1. A 1086-bp Spc^R^ cassette was amplified from plasmid pIC333 using primers Spc-F and Spc-R. A 867-bp right flanking region of *sprT* gene (Rightarm) was amplified from RA-YM genomic DNA using primers SprT-F2 and SprT-R2 (introducing a *Sac*I site). The left flanking region, Spc^R^ cassette, and right flanking region were fused by overlap extension PCR using SprT-F1 and SprT-R2. The fused DNA fragment was inserted into pMD18-T to generate pMD18T-Leftarm-Spc-Rightarm, which was digested along with the pRE112 plasmid with *Kpn*I and *Sac*I to obtain the recombinant suicide plasmid pRE112-Leftarm-Spc-Rightarm (pRE112-LSR). This was transformed into *E. coli X* 7213. The transformants were used as the donor strain in conjugal transfer. The mutant strain was selected on TSA containing 100 μg/mL Spc. The *sprT* gene deletion mutant strain (Δ*sprT*) was confirmed by PCR.

The shuttle plasmid pRES-JX-bla was constructed as previously described (Guo et al., 2017) by adding the putative replication region of the RA plasmid RA-JX to the suicide vector. The promoter and coding sequences of *sprT* were amplified using primers Promoter-sprT1 (introducing a *Kpn*I site) and Promoter-sprT2, SprT-CF, and SprT-CR (introducing a *Sph*I site). The promoter and coding fragments were joined by overlap extension PCR. The product was digested with *Kpn*I and *Sph*I and inserted into pRES-JX-bla to generate plasmid pRES-JX-bla-sprT, which was transferred by conjugation into the mutant strain RA-YMΔ*sprT*. Transconjugants were screened on TSA supplemented with ampicillin and verified by PCR.

### Biochemical Characterization of Mutant Strain Δ*sprT* and Complemented Strain CΔ*sprT*

Growth curves were generated for mutant strain Δ*sprT*, complemented strain CΔ*sprT*, and wild-type strain RA-YM to determine whether *sprT* deletion influenced bacterial growth. The three strains were grown for about 48 h at 37°C in 5% CO_2_ on TSA plates and were inoculated into TSB. At mid-exponential phase, the culture was diluted with fresh medium to an optical density at 600 nm (OD_600_) of 1.0, and equal amounts of each bacterial culture were transferred to fresh TSB medium at a ratio of 1:1000 (v/v) and cultured at 37°C with shaking at 200 rpm. OD_600_ was measured at 1 h intervals for 20 h using a spectrophotometer (Bio-Rad, Hercules, CA, United States).

Biochemical testing of the three strains was carried out using bacterial biochemical tubes (Hope Bio, Qingdao, China) with glucose, arabinose, sucrose, citrate, hydrogen sulfide, nitrate, carbamide, and gelatin. To visually characterize the presence of liquefied gelatin, RA-YM, Δ*sprT*, and CΔ*sprT* cells were inoculated into TSB overnight. Cultures (5 μL) were spotted on nutrient gelatin plates and cultivated at 37°C for 24 h. Then, 2–3 mL acid mercuric chloride was added to the plates and color development was assessed after 5 min. Cells capable of liquefying gelatin, were identified by a transparent halo around the bacterial lawn (Frazier, 1926; Mcdade and Weaver, 1959; Thomas et al., 2014).

### Serum Survival Assay

Blood was drawn from 12 healthy 30-day-old Cherry Valley ducks without anti-RA antibody and immediately placed on ice. After 2 h, the clotted blood was centrifuged at 2000 × *g* and 4°C for 10 min. The serum was collected as normal duck serum and stored at -80°C until use.

For the serum survival assay, mutant strain Δ*sprT*, complemented strain CΔ*sprT*, and wild-type strain RA-YM were cultured in TSB to an OD_600_ of 0.8. The cells were washed and resuspended to 10^6^ CFU/mL in HBSS (Gibco, Grand Island, NY, United States) with 0.15 mM calcium and 1 mM magnesium (HBSS^++^). After incubation at 37°C for 30 min with or without duck serum, 10-fold serial dilutions of the mixture were spread onto TSA plates. Colonies were counted after incubation for 48 h. The serum survival assay was performed with bacteria resuspended in HBSS^++^ to prevent replication so that the viability of all strains could be evaluated (Rosadini et al., 2013). The reaction was also performed in the presence or absence of 10 mM Mg^2+^ EGTA to block the classical complement and lectin pathways and selectively activate the alternative complement pathway. Heat-inactivated serum used in this assay was generated by incubating normal duck serum at 56°C for 30 min. The survival rate was calculated as follows: (number of cells that survived serum treatment/number of cells that survived control treatment) × 100%. Differences among groups were evaluated by analysis of variance using SPSS v.18.0 software (SPSS Inc., Chicago, IL, United States). Data are presented as mean ± standard error of the mean, and statistical significance was determined with the Student’s *t-*test. A *P*-value of <0.05 was defined as statistically significant, and *P*-value of <0.01 was defined as extremely significant.

### Gelatin Enzyme Spectrum Assay

Gelatin zymography was used to evaluate the difference in gelatinase secretion between mutant strain Δ*sprT*, CΔ*sprT* and wild-type strain RA-YM as previously described (Pickett et al., 1991; Toth and Fridman, 2001). Briefly, cells were grown to mid-log phase in TSB at 37°C with shaking, then centrifuged at 10,000 × *g* for 15 min. The culture medium was passed through a 0.22 μm polyvinylidene difluoride filter to remove residual cells, and the supernatant was precipitated with 70% (v/v) saturated ammonium sulfate solution. The precipitate was dissolved with phosphate buffer and samples were resolved by SDS-PAGE on a 10% polyacrylamide gel copolymerized with gelatin (1%) as the substrate at 4°C. The gel was washed for 2 min, proteins were renatured by incubation for 1 h at room temperature in 2.5% Triton X-100 solution, and then incubated at 37°C for 18–20 h in 50 mmol/L Tris-HCl buffer (pH 7.4) containing 5 mmol/L CaCl_2_. The gel was stained with 0.05% Coomassie Brilliant Blue R-250 and then destained with 30% methanol and 10% acetic acid. Gelatinolytic activity was detected as unstained bands against the background of Coomassie-stained gelatin.

### Liquid Chromatography Electrospray Ionization Tandem Mass Spectrometry (LC–MS/MS) Analysis

To detect proteins involved in gelatin hydrolysis, bands that differed between the RA-YM and Δ*sprT* gels were analyzed by LC-MS/MS. The bands were excised and digested overnight in 12.5 ng/μL trypsin in 25 mM NH_4_HCO_3_. The peptides were extracted three times with 60% acetonitrile/0.1% trifluoroacetic acid, and the extracts were pooled and dried completely in a vacuum centrifuge.

Experiments were performed on a Q Exactive mass spectrometer coupled to Easy nLC (Thermo Fisher Scientific, Waltham, MA, United States). A 6 μL volume of each fraction was injected for nanoLC-MS/MS analysis. The peptide mixture (5 μg) was loaded onto a C18 reversed-phase Easy Column (length, 10 cm; inner diameter, 75 μm; and 3 μm resin) (Thermo Fisher Scientific) in buffer A (0.1% formic acid) and separated with a linear gradient of buffer B (80% acetonitrile and 0.1% formic acid) at a flow rate of 250 nL/min controlled by IntelliFlow technology over 140 min. MS data were acquired using a data-dependent top 10 method by dynamically selecting the most abundant precursor ions from the survey scan (300–1800 m/z) for higher-energy collisional dissociation fragmentation. Determination of the target value was based on predictive Automatic Gain Control. The duration of dynamic exclusion was 60 s. Survey scans were acquired at a resolution of 70,000 at m/z 200, and the resolution for HCD spectra was set to 17,500 at m/z 200. Normalized collision energy was 30 eV and the underfill ratio, which specifies the minimum percentage of the target value likely to be reached at maximum fill time, was defined as 0.1%. The instrument was run with peptide recognition mode enabled.

MS/MS spectra were searched using MASCOT engine v.2.2 (Matrix Science, London, United Kingdom) against the non-redundant International Protein Index *Arabidopsis* sequence database v.3.85 (released September 2011; 39,679 sequences) from the European Bioinformatics Institute^1^. The following options were used for protein identification: peptide mass tolerance = 20 ppm, MS/MS tolerance = 0.1 Da, enzyme = trypsin, missed cleavage = 2, fixed modification: carbamidomethyl (C); and variable modification: oxidation (M).

### Expression and Purification of Recombinant RAYM_01812 Protein and Zymography

The coding sequence of the *RAYM_01812* gene (without the signal peptide) was amplified using primers P01812-1 (introducing a *BamH*I site) and P01812-2 (introducing an *Xho*I site). The amplified fragment and vector pGEX-6P-1 were digested with *BamH*I and *Xho*I and ligated to generate the expression vector pGEX-6P-1_01812, which was transformed into *E. coli* BL21 (DE3). Recombinant RAYM_01812 protein was purified as previously described (Ksiazek et al., 2015a). Transformed *E. coli* was grown in Luria-Bertani medium at 37°C to an OD_600_ of 0.8, and isopropyl β-D-1-thiogalactopyranoside was added to a final concentration of 0.2 mM. After cultivation at 25°C for 6 h, cells were harvested by centrifugation and resuspended in sodium phosphate buffer (50 mM, pH7.5). Cells were disrupted twice in a French press at 100 MPa. After centrifugation at 6000 × *g* and 4°C for 15 min, the supernatant was separated from the cell pellet and filtered through a 0.45 μm pore size polyvinylidene difluoride membrane to remove residual cell debris. The protein fraction in the supernatant was purified using an ÄKTA purifier instrument and GST Trap HP crude affinity chromatography column (GST Trap FF, GE, China). The protein was eluted using Tris-HCl (50 mM, pH 8.0) containing 10 mM reduced glutathione. The purified RAYM_01812 protein was incubated at 37°C for 0.5, 1, 4, 8, 24, and 48 h. Protein purity was evaluated by 12% SDS-PAGE and Coomassie staining.

Gelatin zymography of the purified RAYM_01812 protein was performed as previously described (Ksiazek et al., 2015a). The protein was incubated at 37°C for 0.5, 1, 4, 8, 24, and 48 h, then mixed with non-reducing SDS sample buffer (1:1) and incubated at 20°C for 15 min. The mixture was resolved by 10% SDS-PAGE on gels containing gelatin at a final concentration of 1 mg/mL at 4°C. The following operations were analogous to those performed in the gelatin enzyme spectrum experiment. Gelatinolytic activity was visible unstained zones against a blue background.

### Evaluation of Virulence of Mutant Strain Δ*sprT* and Complemented Strain CΔ*sprT in Vivo*

Cherry Valley ducklings (10 days old) were obtained from Chunjiang Duck Farm (Wuhan, China) and allowed to adapt to the environment under controlled temperature (30°C) for 2 days. The ducklings had free access to food and water. The experiments were approved by the Institutional Animal Experiment Committee of the Veterinary Faculty of Huazhong Agriculture University.

Ducklings (12 days old) were randomly divided into 18 groups (*n* = 10 per group) and challenged by intramuscular injection of bacteria at 1.0 × 10^4^, 1.0 × 10^5^, 1.0 × 10^6^, 1.0 × 10^7^, 1.0 × 10^8^, or 1.0 × 10^9^ CFU of mutant strain Δ*sprT*, complemented strain CΔ*sprT*, or wild-type strain RA-YM. The mortality of the ducklings was monitored for 1 week post infection. The LD_50_ was calculated using an improved Karber’s method (Irwin and Cheeseman, 1939).

To evaluate bacterial invasion into organs, we compared bacterial load in the blood and target organs (spleen, liver, heart, and brain) and examined pathological lesions. Cherry Valley ducklings (12 days old) were randomly divided into two groups (*n* = 15 per group) and injected intramuscularly with 1 × 10^7^ CFU bacteria (Δ*sprT* or RA-YM). The organs and blood were collected 24 or 48 h post infection and diluted appropriately. Colonies were counted with the plate pouring method. Data are presented as mean ± standard error of the mean, and statistical significance was determined with the Student’s *t-*test. A *P*-value of <0.05 was defined as statistically significant, and *P*-value of <0.01 was defined as extremely significant. Spleen, liver, heart, and brain tissue sections were immersed in 10% formalin solution for histopathological analyses.

### Vaccination and Challenge Studies

Cherry Valley ducklings (7 days old) were randomly divided into five groups (*n* = 20 per group). Groups 1–3 received a single injection of the mutant strain Δ*sprT* at doses of 4 × 10^5^, 2 × 10^6^, or 1 × 10^7^ CFU. Group 4 was injected with phosphate-buffered saline. Group 5 was injected with trivalent RA oil-inactivated vaccine at the recommended dose (1 × 10^10^ CFU/mL, 0.5 mL) (Tianbang, Chengdu, China). Groups 4 and 5 were served as negative and positive controls, respectively. On day 14 post-immunization, the ducks in each group were injected with 2 LD_50_ or 10 LD_50_ of RA-YM to evaluate the level of protection conferred by Δ*sprT*. The protection rate was calculated using the following formula: [1 - (dead ducklings per group/total ducklings per group)] × 100.

## Results

### Construction and Characterization of Mutant Strain Δ*sprT* and Complemented Strain CΔ*sprT*

**FIGURE 1:**
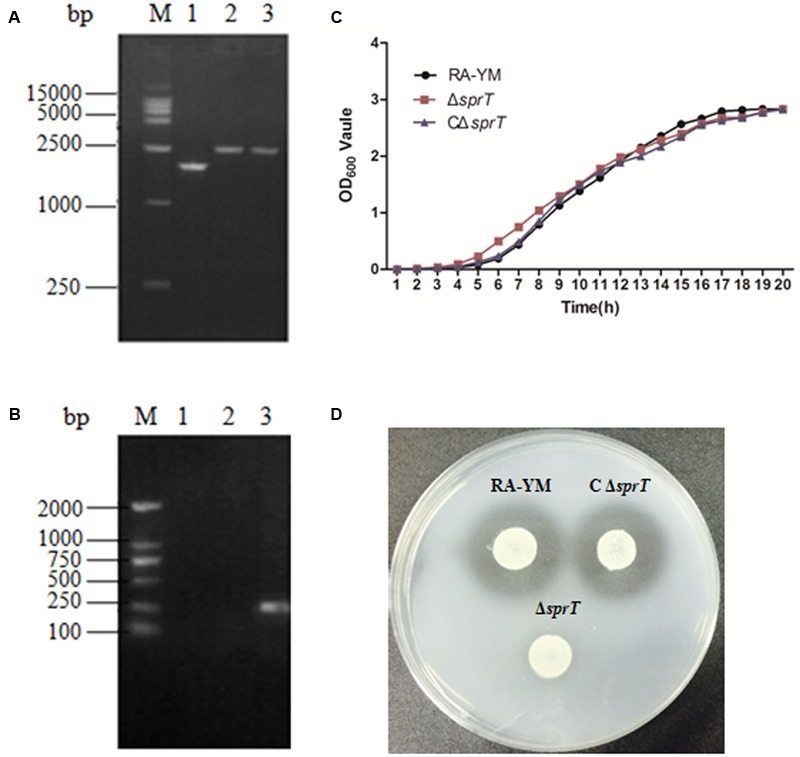
Identification and characterization of mutant strain Δ*sprT* and complemented strain CΔ*sprT*. **(A)** PCR amplification of mutant strain Δ*sprT* and wild-type strain RA-YM using primers SprT-F1 and SprT-R2. Lane M: DL2000 DNA marker; lane 1: Leftarm-Rightarm DNA fragment (1752-bp) from the wild type; lane 2: Leftarm-Spc-Rightarm DNA fragment (2538-bp) from Δ*sprT*; lane 3: pRE-LSR (positive control; 2538-bp). **(B)** PCR amplification of the deleted part of the *sprT* gene using primers SprT-IS and SprT-IR. Lane M: DL2000 DNA marker; lane 1: pRE-LSR (positive control) no fragment was amplified; lane 2: Δ*sprT*—no fragment was amplified; lane 3: RA-YM (300-bp). **(C)** Growth curves for RA-YM, Δ*sprT* and CΔ*sprT*. **(D)** Liquefied gelatin following cultivation of RA-YM, Δ*sprT*, and *C*Δ*sprT* on a nutrient gelatin plate.

The deletion of the *sprT* gene after allelic exchange was confirmed by PCR amplification of the smaller fragment (1752-bp) in RA-YM as compared to the larger Leftarm-Spc-Rightarm fragment (2538-bp) in RA-YMΔ*sprT* (**Figure 1A**). The deleted part of the *sprT* gene (300-bp) was not detected (**Figure 1B**). The identity of the complemented strain was also confirmed by PCR.

There were no differences in biological characteristics between wild-type RA-YM, mutant Δ*sprT*, and complemented CΔ*sprT* strains except in the early logarithmic growth phase, with Δ*sprT* exhibiting more rapid growth as compared to RA-YM, which had a similar growth rate as CΔ*sprT* (**Figure 1C**). In biochemical tests, there was no difference among Δ*sprT*, RA-YM, and CΔ*sprT* except in terms of gelatin liquefaction. All three strains showed negative test results for glucose, arabinose, sucrose, citrate, hydrogen sulfide, nitrate, and carbamide. RA-YM and CΔ*sprT* were able to liquefy gelatin whereas Δ*sprT* was not. The capacity of different strains to liquefy gelatin on nutrient gelatin plates was in accordance with the results of biochemical tests. As shown in **Figure 1D**, there were transparent zones around RA-YM and CΔ*sprT*, but not around Δ*sprT*, demonstrating that RA-YM and CΔ*sprT* were able to liquefy gelatin whereas Δ*sprT* was not.

### Δ*sprT* Mutant Exhibit Increased Sensitivity to Killing by Duck Serum

**FIGURE 2:**
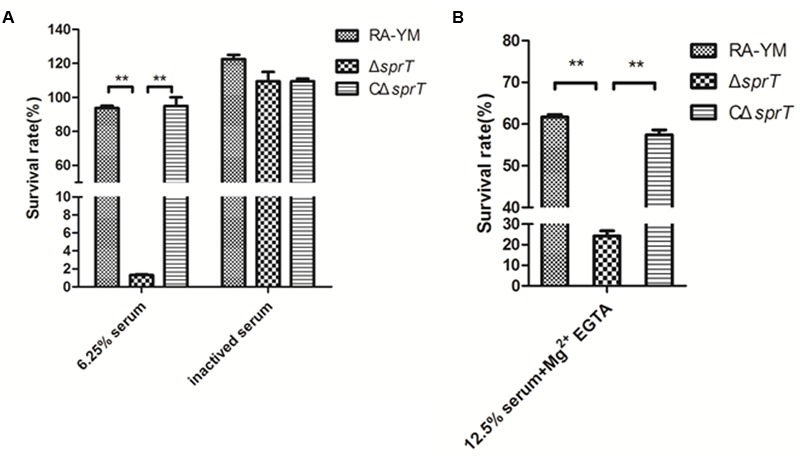
Serum survival of wild-type strain RA-YM, mutant strain Δ*sprT*, and complemented strain CΔ*sprT*. **(A)** Effect of *sprT* mutation on resistance of RA to duck serum. Strains were treated with 6.25% normal duck serum for 30 min at 37°C and plated for survival analysis. **(B)** Effect of *sprT* mutation on resistance of RA to alternative complement pathway-mediated killing. Cells were treated with 12.5% normal duck serum in the presence of Mg^2+^ EGTA for 30 min at 37°C and plated for survival analysis. Data were analyzed with Student’s *t-*test (^∗^*P* < 0.05, ^∗∗^*P* < 0.01).

The serum survival assay was carried out to ascertain the ability of the mutant strain Δ*sprT* to resist complement-mediated killing as compared to the wild-type strain RA-YM. To exclude potential effects of variable growth rates among strains, the assay was performed with the cells resuspended in HBSS^++^, which prevents replication without affecting viability (data not shown). A range of serum concentrations was evaluated for wild-type strain RA-YM; the survival rate of RA-YM was 93.8% in 6.25% normal duck serum. In contrast, the survival rate of Δ*sprT* was 1.3% in 6.25% normal duck serum, whereas that of complemented strain CΔ*sprT* was 95% in 6.25% normal duck serum, which was comparable to the wild-type value (**Figure 2A**). Heat inactivation abrogated the bactericidal effect of serum in Δ*sprT*, consistent with complement-mediated killing. Together, these results indicate that the T9SS is required for complement resistance in wild-type RA cells.

The Δ*sprT* mutants were examined for their sensitivity to alternative complement pathway activation. Strains were treated with normal duck serum in buffer in the presence or absence of 10 mM Mg^2+^ EGTA, which inhibits classical/mannose-binding lectin pathway activation, and then examined for survival. Incubation with Mg^2+^ EGTA-containing buffer alone did not affect the viability of any of the strains (data not shown). When cells were incubated in 12.5% normal duck serum in the presence of Mg^2+^ EGTA, the survival rates of RA-YM Δ*sprT*, and CΔ*sprT* were 61.7, 24.3, and 57.4%, respectively (**Figure 2B**). Taken together, these data indicate that the proteins secreted by T9SS suppress the alternative complement pathway in RA, possibly by binding factor H.

### Gelatin Zymography Assay and LC–MS/MS Analysis

**FIGURE 3:**
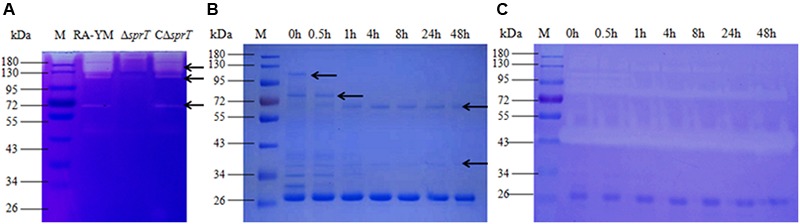
**(A)** Gelatin zymography assay of wild-type strain RA-YM, mutant strain Δ*sprT*, and complemented strain CΔ*sprT*. Regions of clearing reflect protease activity. Black arrows indicate distinct protease activity present in RA-YM and Δ*sprT*. These bands were excised for identification by MS. **(B)** SDS-PAGE analysis of recombinant RAYM_01812 protein incubated at 37°C for different times. M: protein marker; lane 1: purified GST-RAYM_01812 fusion protein; lanes 2–7: purified GST-RAYM_01812 fusion protein incubated at 37°C for 0.5 h (lane 2), 1 h (lane 3), 4 h (lane 4), 8 h (lane 5), 24 h (lane 6), and 48 h (lane 7). **(C)** Ability of purified RAYM_01812 protein to degrade gelatin after incubation at 37°C for different times. M: protein marker; lane 1: purified GST-RAYM_01812 fusion protein; lanes 2–7: gelatin zymography analysis of GST-01812 fusion protein incubated at 37°C for 0.5 h (lane 2), 1 h (lane 3), 4 h (lane 4), 8 h (lane 5), 24 h (lane 6), and 48 h (lane 7).

A gelatin zymography assay was performed to compare gelatinase secretion between mutant strain Δ*sprT* and wild-type strain RA-YM (**Figure 3A**). Distinct bands in the RA-YM culture supernatant were excised and analyzed by MS. The raw data were converted to XML files for an MS/MS ion search using Mascot and were assigned to the protein sequences of RA (strain ATCC 11845/DSM 15868/JCM 9532/NCTC 11014), as annotated by the Uniprot database. Positive hits were accepted with a Mascot score of at least 20. A total of 52 proteins were identified (Supplementary Table S1). T9SS proteins were predicted by running a Function Search analysis of the conserved CTDs of the TIGRfam families TIGR04131 and TIGR04183 (Sato et al., 2013; Kharade and McBride, 2014).

Among the 52 proteins, seven had CTDs belonging to TIGR04183 or TIGR04131, and may therefore be secreted by the T9SS (**Table 2**). RAYM_09380 and RAYM_02622 were hypothetical proteins and RAYM_05530 had a periplasmic ligand-binding sensor domain related to signal transduction. The four other proteins all had a Por_Secre_tail, but their functions differed according to their specific structural domains. RAYM_08375, containing a polycystic kidney disease (PKD) and two Sialidase_non-viral domains, was predicted to encode glycosyl hydrolase. RAYM_04099, containing a Peptidases_S8_Kp43_protease domain, encodes peptidase S8/S53 subtilisin kexin sedolisin. RAYM_03382, containing a metallophosphatase superfamily and calcineurin-like phosphoesterase domains, was predicted to encode phosphohydrolases. RAYM_01812, also containing Peptidases_S8_S53 superfamily and peptidase_S8 domains, encodes a subtilisin-like serine protease.

**Table 2:** C-terminal domains (CTDs) of proteins identified in zymogram gels of RA-YM.

Locus tag	Description	MW (KDa)	CTD present	TIGRfam
RAYM_09380	Hypothetical protein	131.7	+	TIGR04131
RAYM_03382	Phosphohydrolases	68.2	+	TIGR04183
RAYM_04099	Subtilisin-like serine protease	161.5	+	TIGR04183
RAYM_08375	Glycosyl hydrolase	117.3	+	TIGR04183
RAYM_01812	Subtilisin-like serine protease	62.3/77.8	+	TIGR04183
RAYM_05530	Immunoreactive 84 kDa antigen PG93	83.1	+	TIGR04183
RAYM_02622	Hypothetical protein	117.1	+	TIGR04183

RAYM_01812 was expressed as a fusion protein with a molecular mass of 102 kDa and the capacity for autoproteolytic cleavage (**Figure 3B**). The full-length recombinant RAYM_01812 protein (without GST label) had a mass of 76 kDa. Following removal of the NTP, preCTD and CTD regions, the active protease comprising only CD regions was left with a molecular mass of 38 kDa. Gelatin zymography analysis of purified RAYM_01812 revealed that three forms of the protein were capable of degrading gelatin (**Figure 3C**).

### Virulence of Mutant Strain Δ*sprT* and Complemented Strain CΔ*sprT in Vivo*

The LD_50_ for the mutant strain Δ*sprT* was 1.61 × 10^9^ CFU, which was 4.21 × 10^4^-fold lower than that of the parent strain RA-YM (3.82 × 10^4^ CFU) and of CΔ*sprT* (4.37 × 10^4^ CFU). This indicated that the virulence of Δ*sprT* was potently suppressed, an effect that was rescued by complementation. Since the difference between RA-YM and CΔ*sprT* was negligible, in subsequent experiments we compared only RA-YM and Δ*sprT*.

**FIGURE 4:**
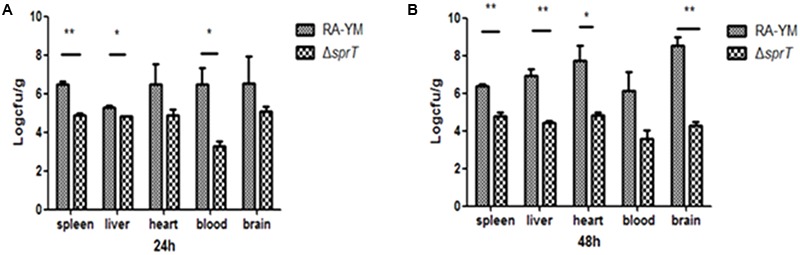
Bacterial load in blood and organs of ducklings infected with wild-type strain RA-YM or mutant strain Δ*sprT*. Tissue burden in groups infected with RA-YM or Δ*sprT* 24 h **(A)** and 48 h **(B)** post challenge. Data represent mean ± standard deviation of five animals. Data were analyzed with Student’s *t-*test (^∗^*P* < 0.05, ^∗∗^*P* < 0.01).

**FIGURE 5:**
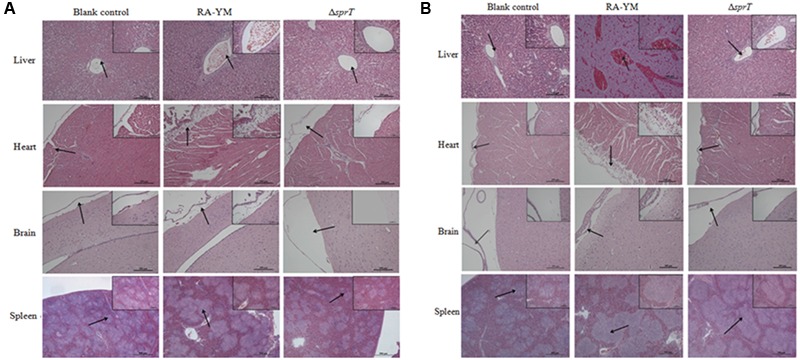
Histopathological analysis of ducklings infected with wild-type strain RA-YM or mutant strain Δ*sprT*. Tissue specimens were analyzed 24 h **(A)** and 48 h **(B)** post challenge.

To examine the invasiveness of the bacteria in greater detail, bacterial load in the blood, spleen, liver, heart, and brain was quantified. The bacterial burden of Δ*sprT* in the blood, spleen, and liver was much lower than that of RA-YM at 24 and 48 h post challenge (**Figure 4**). We also compared tissue damage caused by RA-YM and Δ*sprT*. In the RA-YM group, there was obvious congestion in the hepatic sinusoid and central vein, fatty degeneration of hepatocytes, a large quantity epicardial fibrin exudate, and inflammatory cells; notably, the latter two were present also in the subarachnoid space. Additionally, lymphocyte proliferation of splenic white pulp was also observed. This phenotype was greatly attenuated in the Δ*sprT* and negative control groups at 24 h (**Figure 5A**) and 48 h (**Figure 5B**) post challenge.

### Protective Effect of Vaccination with Mutant Strain Δ*sprT* against Virulent RA-YM

Finally, we evaluated whether the mutant strain Δ*sprT* could be used as a live attenuated vaccine. Ducks were administered 2 LD_50_ and 10 LD_50_ of virulent RA-YM by intramuscular injection 14 days after vaccination. At 24 h post-injection, clinical symptoms and death were observed in Group 4, whereas ducks in Groups 1–3 that were vaccinated with Δ*sprT* showed only mild symptoms. When Δ*sprT* was injected at a dose of 1 × 10^7^ CFU per duck (Group 3), immune protective ratios were 100 and 88.9% as compared to 80 and 70%, respectively, in the vaccine group (**Table 3**).

**Table 3:** Immune effect of the mutant strain Δ*sprT.*

Group	Inoculation type	Survival of ducks/total ducks		Protection rates (%)	
		2 LD_50_	10 LD_50_	2 LD_50_	10 LD_50_
1	Δ*sprT* (4 × 10^5^ CFU)	6/10	7/10	60	70
2	Δ*sprT* (2 × 10^6^ CFU)	8/10	6/8	80	75
3	Δ*sprT* (1 × 10^7^ CFU)	9/9	8/9	100	88.9
4	PBS (negative control)	1/10	0/10	10	0
5	Oil-inactivated vaccine (5 × 10^9^ CFU)	8/10	7/10	80	70

## Discussion

Specialized protein secretion systems in many pathogenic bacteria comprise virulence mechanisms for the secretion of extracellular enzymes, proteases, and virulence factors that destroy host cells and cause tissue necrosis. Eight such systems have been identified to date in Gram-negative bacteria (Economou et al., 2006). T1SS, T3SS, T4SS, and T6SS transport proteins from the cytoplasm across both membranes of the cell, whereas T2SS, T5SS, T8SS (the extracellular nucleation-precipitation pathway involved in curli biogenesis), and the chaperone-usher pathway (involved in pilus assembly) facilitate secretion across the outer membrane only. T9SS, which is also known as Por secretion system, is present in most genera and species of the phylum *Bacteroidetes*, including *F. johnsoniae, P. gingivalis, T. forsythia*, and the poultry pathogen RA (Mcbride and Zhu, 2013). *P. gingivalis* T9SS is involved in the secretion of virulence factors, such as Arg-gingipain, Lys-gingipain, and Skp-protein, which damage human tissues and dysregulate the immune response (Nakayama et al., 1995; Shi et al., 1999; Sato et al., 2013; Taguchi et al., 2015). *T. forsythia* also uses this pathway to disseminate proteins, such as KLIKK proteases (Narita et al., 2014; Ksiazek et al., 2015b). In this study, the virulence of the mutant strain Δ*sprT* was attenuated by more than 42,000-fold and bacterial load in the blood, spleen, brain, heart, and liver of ducks infected with Δ*sprT* were significantly reduced as compared to those of RA-YM-infected ducks. Moreover, this tendency was confirmed by histopathological results. Thus, our findings confirm that T9SS plays a critical role in the pathogenicity of RA. We also show that Δ*sprT* administration effectively protected the ducks from challenge with virulent RA-YM strains, suggesting that the mutant strain can be used as an attenuated vaccine target or live vaccine vector for controlling septicemia anserum exsudativa in ducks.

Bacterial virulence factors are secreted proteins that have pathogenic effects. PorT was reported to be involved in the secretion of gingipains and the mutant showed decreased Arg- and Lys-gingipains activities in both the whole-cell and supernatant fractions (Sato et al., 2005; Nguyen et al., 2009). Deleting *porT* in *T. forsythia* results in the lack of an S-layer, which functions as a protective coat, external sieve, and ion trap (Narita et al., 2014; Tomek et al., 2014). The *F. johnsoniae sprT* mutant is defective in extracellular chitinase activity (Sato et al., 2010). In RA, deletion of *sprT* yielded cells that failed to liquefy gelatin; this phenotype was rescued by complementation with wild-type *sprT*.

**FIGURE 6:**
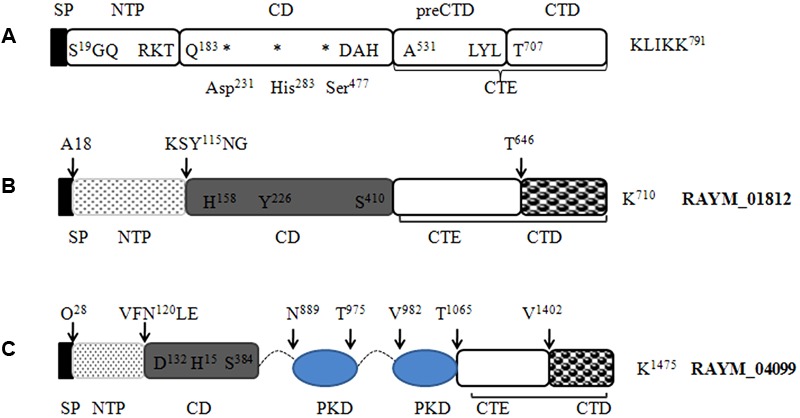
Predicted protein domain structure of the S8 family of serine proteases in RA. Predicted structures of mirolase **(A)** (Ksiazek et al., 2015a), RAYM_01812 **(B)**, and RAYM_04099 **(C)**. Signal peptides were predicted using the Signal IP 3.0 Server.

Our LC-MS/MS analysis revealed that RAYM_01812 and RAYM_04099 were secreted by the T9SS. A comparison of their sequences revealed a domain structure that included a typical N-terminal signal peptide, subtilisin-like protease catalytic domain and C-terminal extension (**Figure 6**) (Ksiazek et al., 2015a). RAYM_01812 and RAYM_04099 showed 32 and 20% identity with *T. forsythia* mirolase, a subtilisin-like serine protease (Ksiazek et al., 2015a); and 34 and 40% identity with *Pseudoalteromonas* sp. SM9913 deseasin MCP-01, a collagenolytic, cold-adapted serine protease (Chen et al., 2007). The PKD domain of RAYM_04099 was reported to be a collagen-binding domain that could improve the collagenolytic efficiency of the catalytic domain (Wang et al., 2010). These results indicate that *RAYM_01812* and *RAYM_04099* encode serine proteases. The conserved catalytic residues of the Peptidases_S8_S53 superfamily domain may be responsible for the hydrolysis of gelatin, which was confirmed for recombinant protein RAYM_01812 by gel electrophoresis and zymography. Gelatinolytic activity is the most significant biochemical property in virulent RA; this is the first report of a protease related to gelatin degradation and of subtilisin-like serine proteases in the culture supernatant of RA. In some Gram-negative bacteria, special proteinases contribute to host invasion (Backert and Meyer, 2006). For instance, gelatinase can degrade host collagen and generate nutrients for the pathogen, thereby enhancing the pathogen’s aggressiveness (Corbel et al., 2002). These proteins are either released into the extracellular matrix or directly injected into host cells (Juhas et al., 2008). Previous studies have shown that in *Enterococcus*, the gene encoding gelatinase was a virulence factor whose expression could directly influence bacterial pathogenicity (Sifri et al., 2002; Engelbert et al., 2004), and a serine protease known as dentilisin in *Treponema denticola* has been implicated in complement evasion by C3 cleavage (Yamazaki et al., 2006). Mirolase, a subtilisin-like serine protease, contributes to *T. forsythia* pathogenicity by degrading fibrinogen, hemoglobin, and the antimicrobial peptide LL-37 (Ksiazek et al., 2015a).

To induce bacteremia, pathogenic bacteria must evade serum killing, which is mainly mediated by the complement system. The mechanism of complement resistance in bacteria involves protease digestion of complement components; recruitment of factors such as factor H and C4-binding protein—which inhibit complement activation—to the bacterial cell surface; and polysaccharide-mediated suppression of complement activation. In this study, we showed that SprT protein was required for full serum resistance and for defense against alternative complement pathway activation. Thus, proteins secreted by the T9SS are involved in complement resistance in RA. The Δ*sprT* mutant is likely defective in the secretion of virulence factors into the extracellular environment or in their expression on the cell surface, which facilitate evasion of complement-mediated killing. Additionally, secreted proteins may directly cleave antibodies and complement components or bind complement-inhibiting molecules; indeed, proteolytic inactivation of the complement system is a common strategy for avoiding host immune surveillance mechanisms. A number of studies have shown that proteolytic enzymes such as *T. denticola* dentilisin, *P. gingivalis* gingipains, *T. forsythia* karilysin, and *P. intermedia* interpain are primary weapons of defense against host innate immunity. Additional studies are needed to clarify the role of proteinases such as RAYM_01812 that are secreted through the T9SS in the degradation and functional inactivation of host complement components.

In summary, the results presented here demonstrate that the T9SS—which is involved in the secretion of the subtilisin-like proteases RAYM_01812 and RAYM_04099 with extracellular gelatinase activity—is critical for the virulence of RA. Given that the T9SS of RA is involved in serum resistance, we are now working to identify T9SS substrates and verify their roles in evading the complement system, which can provide insight into the virulence mechanism of this pathogen.

## Author Contributions

YG and DH wrote the manuscript. Construction of the mutant strain Δ*sprT*, its virulence, and immune protection were determined by TW. The complemented strain was constructed by YG. The expression of the recombinant protein RAYM_01812 was performed by JG. Gelatin zymography and LC–MS/MS analysis were performed by DH. YX, XW, SL, ML, ZL, and DB designed and participated to the experiments. ZZ designed and revised the manuscript. All authors read and approved the final manuscript.

## Conflict of Interest Statement

The authors declare that the research was conducted in the absence of any commercial or financial relationships that could be construed as a potential conflict of interest.

## Footnotes

1 http://www.ebi.ac.uk/

## Abbreviations

CFU: Colony-forming units
CTD: C-terminal domain
HBSS: Hank’s balanced salt solution
LC-MS/MS: liquid chromatography electrospray ionization tandem mass spectrometry
LD_50_: median lethal dose
RA: *Riemerella anatipestifer*
SDS-PAGE: sodium dodecyl sulfate-polyacrylamide gel electrophoresis
T9SS: type IX secretion system
TSA: tryptic soy agar
TSB: tryptic soy broth
