# Supplementary material for: Riemerella anatipestifer Type IX Secretion System Is Required for Virulence and Gelatinase Secretion
Source: Front Microbiol. 2017 Dec 19;8:2553. doi: 10.3389/fmicb.2017.02553 (PMC5742166; doi:10.3389/fmicb.2017.02553)
Supplement: Supplementary file 1 [file Presentation_1.PDF]

**Table S1: Proteins identified in zymogram gels of RA-YM**

| Locus tag | Acession | Description | MW(KDa) |
|---|---|---|---|
| *C-terminal-domain-containing proteins* | | | |
| RAYM_09380 | E4TEE3 | hypothetical protein | 131.6 |
| RAYM_03382 | E4T8X7 | PA14 domain protein | 68.2 |
| RAYM_04099 | E4T8U9 | subtilisin-like serine protease | 161.5 |
| RAYM_08375 | E4TA79 | glycosyl hydrolase | 117.3 |
| RAYM_01812 | H8MA42/ J9QYT3/H8MA43 | subtilisin-like serine protease | 62.3/77.8 |
| RAYM_05530 | E4T9B5 | immunoreactive 84 kDa antigen PG93 | 83.1 |
| RAYM_02622 | E4TBI9 | hypothetical protein | 117.1 |
| *Hydrolytic enzymes* | | | |
| RAYM_03989 | E4TAY4 | catalase katb | 58.2 |
| RAYM_09764 | E4T958 | 5-hydroxyisourate hydrolase | 15.9 |
| RAYM_02367 | E4T9X2 | glycine dehydrogenase | 105.1 |
| RAYM_08295 | E4TA95 | peptidase M28 | 57.9 |
| RAYM_04124 | J9R9V1 | UDP-N-acetylglucosamine enolpyruvyl transferase | 104.1 |
| RAYM_02242 | E4T9Z6 | Dipeptidyl peptidase IV | 81.5 |
| RAYM_08330 | E4TA88 | peptidase S46 | 80.6 |
| *TonB-associated outer membrane proteins* | | | |
| RAYM_09500 | E4TAX0 | SusC/RagA family TonB-linked outer membrane protein | 100.7 |
| RAYM_02017 | E4TBP4 | TonB-dependent receptor, plug | 109.4 |
| RAYM_04896 | V9TM77/ V9TJP7/ E4TDA5 | TonB-dependent outer membrane receptor | 100.5 |
| RAYM_01847 | E4TBS7 | putative TonB-dependent outer membrane protein | 81.5 |
| RAYM_00850 | E4TCK6/ J9R5T4 | TonB-dependent receptor plug | 76.1 |

| | | | |
|---|---|---|---|
| RAYM_08790 | E4TE30 | TonB-dependent receptor | 83.5 |
| RAYM_00475 | J9R767 | TonB-dependent receptor | 96.6 |
| RAYM_04144 | H8MCT0 | putative TonB-dependent receptor | 67.1 |
| RAYM_05550 | E4T9B9 | TonB-dependent receptor, putative | 97.0 |
| RAYM_07669 | E4TC35 | TonB-dependent siderophore receptor | 89.1 |
| Outer membrane proteins | | | |
| RAYM_00470 | H8MC56 | cell envelope biogenesis protein OmpA | 121.7 |
| RAYM_07139 | E4TCE1/ H9ZJ07 | OmpA | 52.3 |
| RAYM_06882 | E4T9T1 | Outer membrane hemin receptor | 89.6 |
| *Other proteins* | | | |
| RAYM_04961 | H8MDY2/ H8MDY1 | RHS repeat-associated core domain protein | 171.6/ 167.3 |
| RAYM_09505 | E4TAX1 | RagB/SusD domain protein | 59.0 |
| RAYM_00825 | J9R5T9 | putative flagellar motor protein MotB | 30.4 |
| RAYM_05206 | E4TD46 | Hep_Hag family protein | 48.0 |
| RAYM_02537 | E4TBK6 | putative hemin receptor | 53.3 |
| RAYM_07554 | E4TC58 | TPR repeat-containing protein | 60.1 |
| RAYM_03077 | H8MDB5 | UPF0246 protein YaaA | 27.4 |
| RAYM_01947 | M4T436 | putative lipoprotein | 51.6 |
| *Hypothetical proteins* | | | |
| RAYM_08875 | E4TE47 | hypothetical protein | 104.9 |
| RAYM_01000 | E4TCN6 | hypothetical protein | 60.3 |
| RAYM_08305 | E4TA93 | hypothetical protein | 109.1 |
| RAYM_07629 | E4TC43/J9R1C3 | hypothetical protein | 101.7 |
| RAYM_03422 | E4T8W9 | hypothetical protein | 24.6 |
| RAYM_02022 | E4TBP3 | hypothetical protein | 54.5 |
| RAYM_06000 | E4T9K8 | hypothetical protein | 59.8 |

| | | | |
|---|---|---|---|
| RAYM_01952 | E4TBQ7 | hypothetical protein | 106.9 |
| RAYM_03699 | E4TB34 | hypothetical protein | 40.8 |
| RAYM_08875 | E4TE47 | hypothetical protein | 104.9 |
| RAYM_01655 | H8MDH7 | hypothetical protein | 74.3 |
| RAYM_00570 | E4T8S7 | hypothetical protein | 108.3 |
| RAYM_02382 | E4TBN7 | hypothetical protein | 30.4 |
| RAYM_08325 | E4TA89 | hypothetical protein | 33.2 |
| RAYM_03484 | E4TB76 | hypothetical protein | 19.8 |
| RAYM_04486 | E4TDI3 | hypothetical protein | 48.7 |
| RAY M_05335 | E4T976 | hypothetical protein | 80.6 |
